# A Density Functional Theory (DFT) Perspective on Optical Absorption of Modified Graphene Interacting with the Main Amino Acids of Spider Silk

**DOI:** 10.3390/ijms241512084

**Published:** 2023-07-28

**Authors:** Ali Fransuani Jiménez-González, Juan Manuel Ramírez-de-Arellano, Luis Fernando Magaña Solís

**Affiliations:** 1 Instituto de Física, Universidad Nacional Autónoma de México, Mexico City 01000, Mexico; 2 Tecnologico de Monterrey, Escuela de Ingeniería y Ciencias, Mexico City 14380, Mexico

**Keywords:** graphene, amino acids, DFT, optical properties

## Abstract

We investigated the possible adsorption of each of the main building blocks of spider silk: alanine, glycine, leucine, and proline. This knowledge could help develop new biocompatible materials and favors the creation of new biosensors. We used ab initio density functional theory methods to study the variations in the optical absorption, reflectivity, and band structure of a modified graphene surface interacting with these four molecules. Four modification cases were considered: graphene with vacancies at 5.55% and fluorine, nitrogen, or oxygen doping, also at 5.55%. We found that, among the cases considered, graphene with vacancies is the best candidate to develop optical biosensors to detect C=O amide and differentiate glycine and leucine from alanine and proline in the visible spectrum region. Finally, from the projected density of states, the main changes occur at deep energies. Thus, all modified graphene’s electronic energy band structure undergoes only tiny changes when interacting with amino acids.

## 1. Introduction

At present, developing biosensors to detect antibodies, nucleic acids, and peptides is of great interest to several fields, with potential health, industrial, and environmental applications. Several materials have been studied from silicon [1,2] to different 2D nanostructures or even paper [3,4], but graphene is a promising candidate for the development of these technologies [5,6] due to its interesting thermal [7], high-strength [8,9], electronic, and optical properties [10]. Heteroatom doping of graphene has been explored to enhance or tune the said properties to reach particular objectives, e.g., the enhancement of its electrochemical performance [11] and optical properties [12,13].

Recently, engineered biosensors have provided a high electron transfer between molecules and electrodes. The high electron mobility of graphene, combined with the relatively easy way of doping, makes it an excellent candidate for a molecule sensor. Another favorable feature of graphene is its transparency to the visible spectrum. The large number of states provided by a biomolecule considerably modifies this property, making it useful for optical sensors [14,15].

This work focuses on the interaction between graphene and four amino acids, known to be the main building blocks for spider silk [16]: alanine, glycine, leucine, and proline. Spider silk is an interesting material because of the combination of its elasticity, equivalent to that of rubber, and strength and stiffness, similar to that of steel. Moreover, spider silk is highly sensitive to multiple environmental stimuli such as humidity, temperature, and strain [17].

Investigating the individual interactions of graphene with the amino acids of spider silk could yield a successful blending of graphene and spider silk. It may be necessary to functionalize graphene with peptidic chains to achieve this goal. The new material obtained from this blending may result in a combination of the extraordinary mechanical properties of each material. Ab initio density functional methods provide an excellent tool for such an investigation.

## 2. Results

### 2.1. Optimized Structures

**Figure 1:**
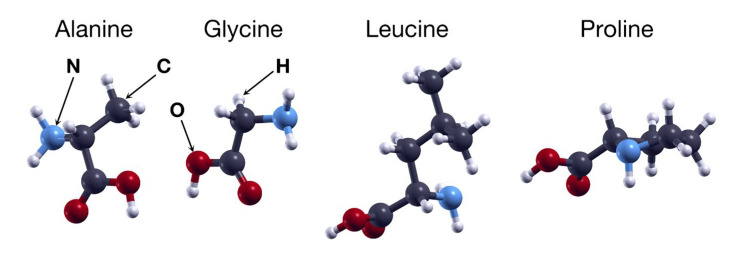
Structures of the four amino acids considered in this work. From left to right: alanine, glycine, leucine, and proline.

**Figure 2:**
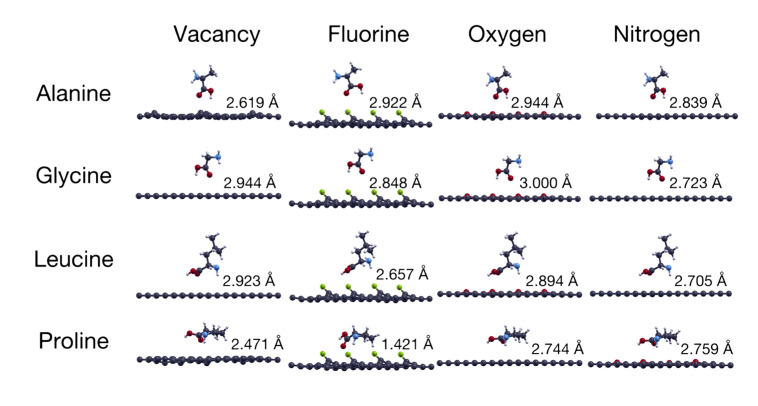
The figure shows the final configurations for the structural optimizations performed on the 16 cases considered. The columns show the four different modified graphene surfaces considered: graphene with vacancies and F-, O-, and N-doped graphene, all at 5.55%. The rows show the interactions of each graphene structure with the four different amino acids considered: alanine, glycine, leucine, and proline. The initial vertical distance between the surface and the amino acid was 3 Å for all cases, while next to each case, the figure shows the final vertical distance of each amino acid and the surface, also in Å.

Figure 1 shows the structures of the four amino acids considered, while Figure 2 shows the 16 optimized structures of the modified graphene studied in this work, including graphene with a vacancy and fluorine, nitrogen, or oxygen doping. The four amino acids were found to be neutrally charged and the initial distance from the graphene plane was 3 Å in all cases. Based on previous studies [14,18], the carboxyl group of alanine, glycine, and leucine had the lowest energy configuration compared with the other functional groups. For the proline, the carbon ring was oriented parallel to the plane of the modified graphene.

Next to each case in Figure 2, we show the final vertical distance between each amino acid and the corresponding graphene surface after performing structural optimizations. Considering again that the initial vertical distance was 3 Å, Figure 2 shows that proline approached the graphene with vacancies the most, as well as the F- and O-doped graphene surfaces. For the N-doped graphene surface, it was leucine that ended up being closest to it. On the other hand, glycine on O-doped graphene showed the least noticeable change in distance, with a variation of 0.0005 Å in its final vertical position, which can be considered to be negligible.

The left column in Figure 2 shows the interactions of the four amino acids with graphene at 5.55% vacancies. We found that proline repelled one of the carbon atoms surrounding the vacancy, probably due to the carbon ring of proline. We found the opposite effect with alanine, which attracted the carbon atoms around the vacancy, causing a deformation on the graphene’s surface. All the other structures left graphene with no significant changes.

**Figure 3:**
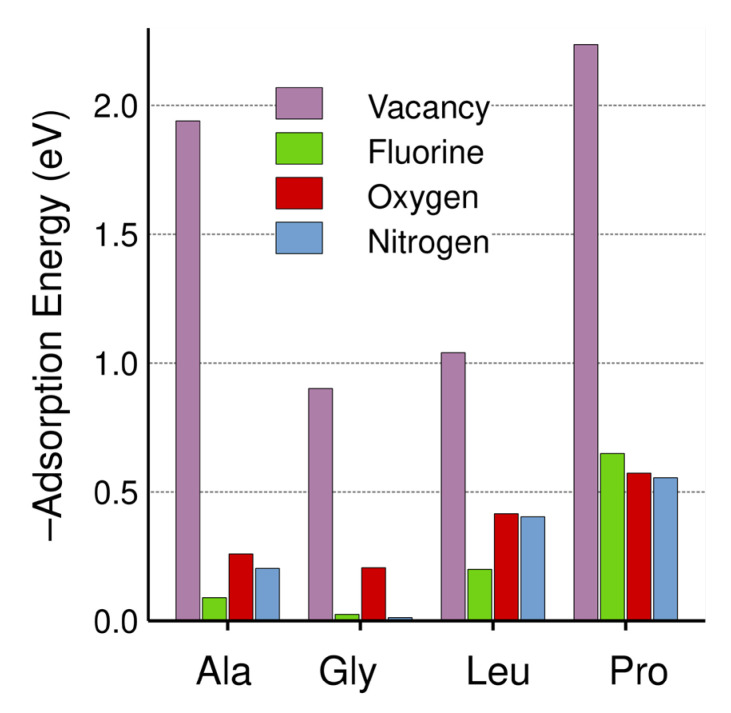
Absorption energies (in eV) as a function of each amino acid for vacant and doped graphene surfaces. All the energies are negative values.

We calculated the adsorption energies using the following equation:

(1) $$E_{\mathrm{ads}}{=E}_{\mathrm{Final}\;\mathrm{structure}}-\left(E_{X}{+E}_{\mathrm{modified}\;\mathrm{graphene}}\right)$$

where *X* = alanine, glycine, leucine, or proline and the modified graphene energy refers to the energy of either graphene with vacancies or the F-, O-, or N-doped graphene surfaces. The results are shown in Figure 3 and Table 1. Glycine interacted least with the graphene structures, while proline interacted the most. This result may have been caused by the π-electrons of the carbon ring. Overall, except for proline, we saw that the adsorption energy increased along with the number of atoms in the amino acid. The interaction between alanine and the graphene surface with vacancies showed the second highest adsorption energy, with the atoms surrounding the vacancy approaching the alanine molecule, causing an increase in the adsorption energy.

We calculated the recovery times from Equation (6) using the values for the adsorption energies shown in Table 1. Figure 3 is a visualization aid for Table 1. It is usually considered that an appropriate value of the recovery time for a proper detection should be in the interval from 0.01 to 100 s, which is equivalent to adsorption energies between −0.64 eV and −0.82 eV [19,20]. Table 2 shows the values obtained for the recovery times for each case considered. From Table 1 and Table 2, we can see that graphene with vacancies was the most suitable for the detection of glycine, while F-doped graphene at the same percentage could be suitable for detecting proline.

The adsorbed molecule should remain on the sensor’s surface for a significant enough time to consider the latter as a suitable detector. In this way, it is possible to measure some convenient properties of the material, searching for changes. However, considerable recovery times could rapidly lead to surface saturation, making the detector unsuitable.

### 2.2. Band Structure

**Figure 4:**
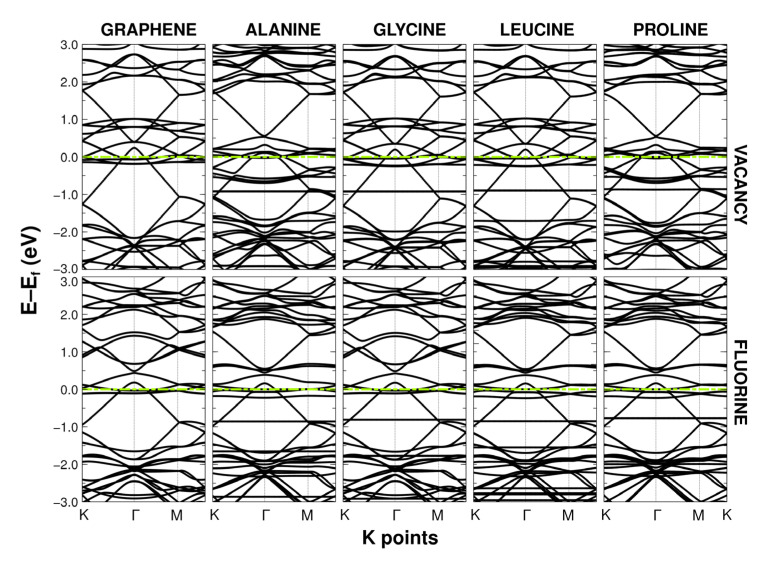
Band structures of graphene with vacancies and F-doped graphene (both at 5.55%) when interacting with alanine, glycine, leucine, and proline. The dashed green line refers to the Fermi energy level.

**Figure 5:**
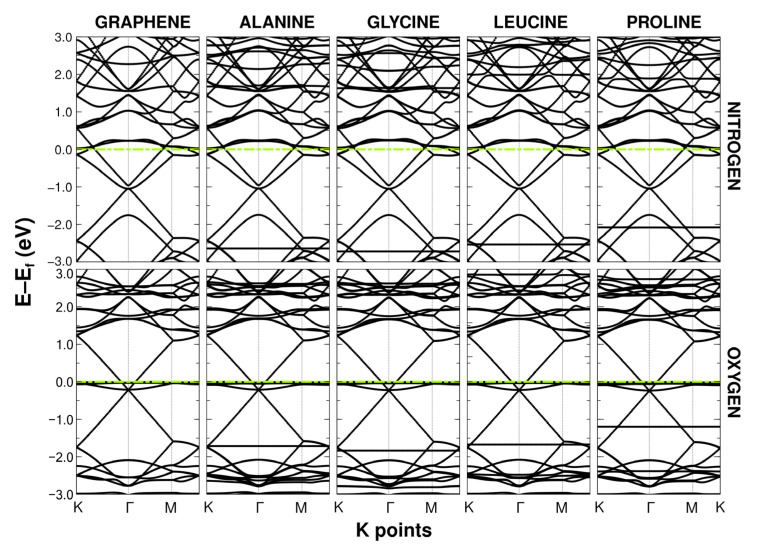
Band structures of N-doped graphene and O-doped graphene (both at 5.55%) when interacting with alanine, glycine, leucine, and proline. The dashed green line refers to the Fermi energy level.

The cell size used showed Brillouin folding, placing the Dirac cone at the gamma point instead of the kappa point. However, as this was an effect of the symmetry and size of the unit cell, it left the results unchanged. In all cases, the presence of the amino acids generated a semi-flat band that could vary in a range from −0.9 eV to −2.7 eV, as seen in Figure 4 and Figure 5. This seemed to imply that the electronic properties of the doped or vacant graphene remained practically the same and with conductive behavior.

### 2.3. PDOS

**Figure 6:**
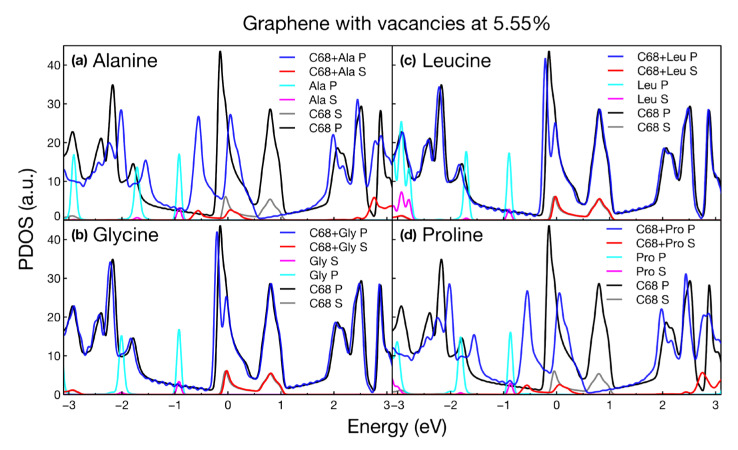
Projected density of states for graphene doped with vacancies at 5.55% interacting with (**a**) alanine, (**b**) glycine, (**c**) leucine, and (**d**) proline. The plot shows the variations in each orbital for the different cases considered.

**Figure 7:**
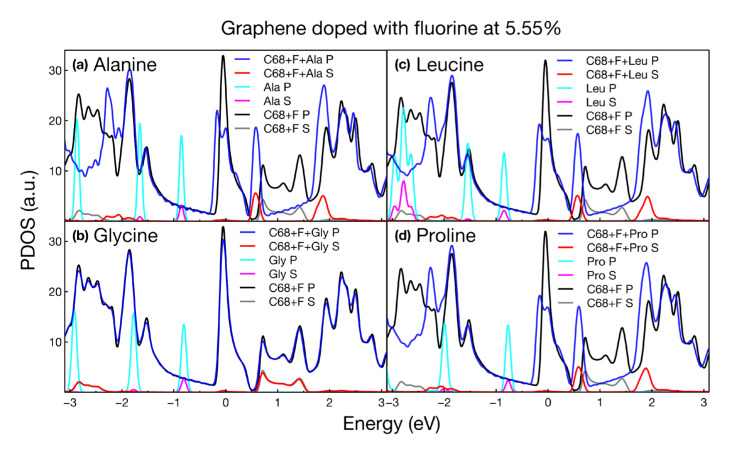
Projected density of states for graphene doped with fluorine at 5.55% interacting with (**a**) alanine, (**b**) glycine, (**c**) leucine, and (**d**) proline.

**Figure 8:**
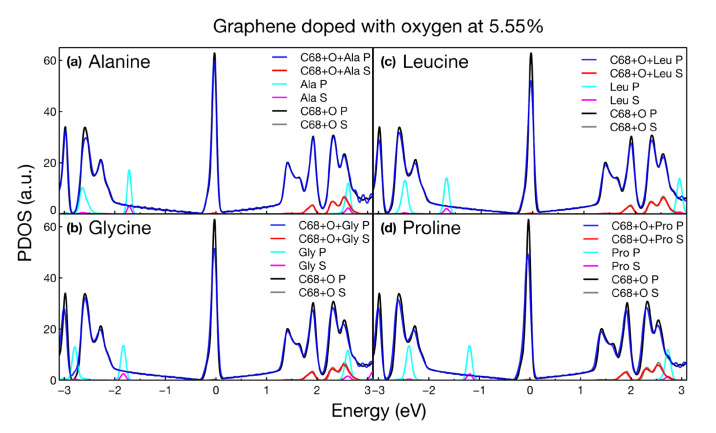
Projected density of states for graphene doped with oxygen at 5.55% interacting with (**a**) alanine, (**b**) glycine, (**c**) leucine, and (**d**) proline.

**Figure 9:**
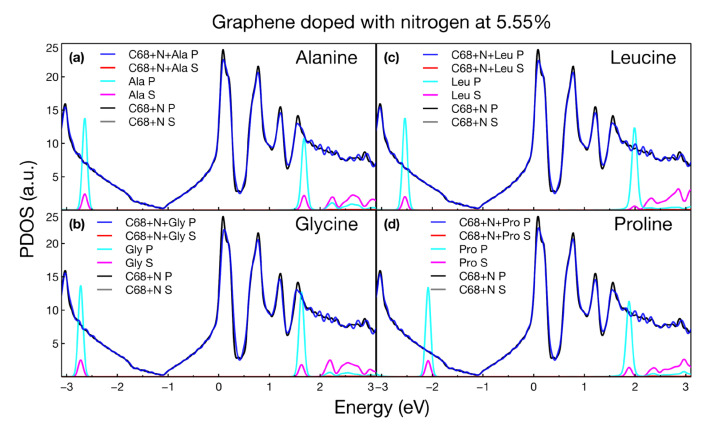
Projected density of states for graphene doped with nitrogen at 5.55% interacting with (**a**) alanine, (**b**) glycine, (**c**) leucine, and (**d**) proline.

We obtained the projected density of states (PDOS) for all the calculated structures. We present the variations in the s and p states of graphene modified with and without amino acids. Also, we show the individual states of each amino acid. For all structures considered, the PDOS plots confirmed the metallic nature of the systems, shown as well in the band structure plots. Figure 6, Figure 7, Figure 8 and Figure 9 show the PDOS for all cases considered.

As can be seen in Figure 6, the effect of alanine and proline on graphene at 5.55% vacancies shifted two peaks to the left: the main peak located at the Fermi level and the peak located at 0.9 eV. These shifts may have been due to the strong interaction between both amino acids and graphene because both have adsorption energy of around 2 eV. The semi-metallic nature of the system was also unaffected. For leucine and glycine, the adsorption was weaker and the states remained almost unchanged.

For F-doped graphene (Figure 7), the changes in the PDOS were very similar for all amino acids, with only glycine keeping the PDOS intact around the Fermi level. Moreover, glycine had the lowest adsorption energy. In the other three cases, the effect of the amino acids reduced the number of states at the Fermi level and the states above the Fermi level from 0.7 to 1.45 eV were condensed into a single peak at 0.55 eV.

Figure 8 and Figure 9 show no modification to the PDOS around the Fermi level upon the interaction of the four amino acids with graphene doped with oxygen and nitrogen, respectively.

The main changes in the PDOS occurred at deeper energies, which modified the optical absorption shown in the next section. In this and the previous section, we only focused on showing the invariance of the electronic properties as the four amino acids interacted with the vacancies and various dopants. We will see that optical activity is the main form of detection and discrimination of amino acids.

The electron transfer (Löwdin charges) was low, with values around 10^−2^, suggesting that only Van der Waals interactions were relevant. We have not reported the Löwdin charge values in this work because of their low-range variance.

### 2.4. Optical Absorption and Reflectivity

**Figure 10:**
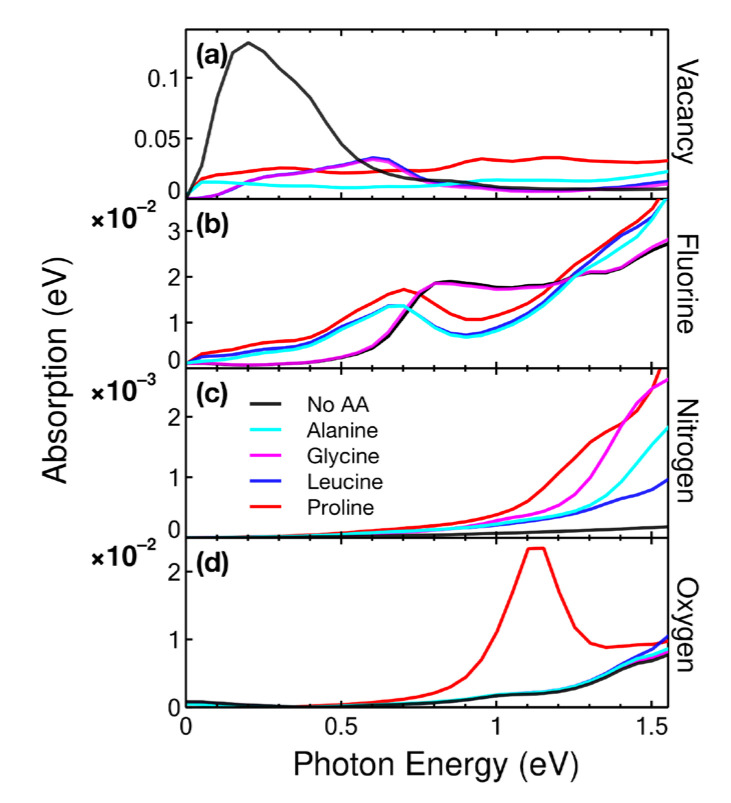
Absorption in the infrared region for the cases considered: (**a**) vacancy, (**b**) F-doped, (**c**) N-doped, and (**d**) O-doped graphene at a concentration of 5.55% interacting with alanine and glycine.

**Figure 11:**
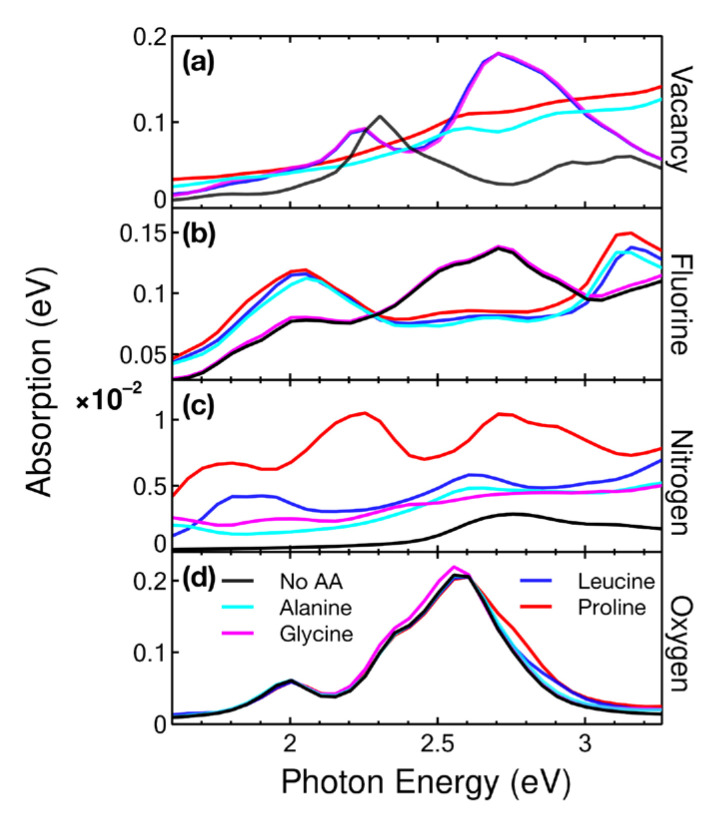
Absorption in the visible region for the cases considered: (**a**) vacancy, (**b**) F-doped, (**c**) N-doped, and (**d**) O-doped graphene at a concentration of 5.55% interacting with alanine, glycine, leucine, and proline.

From the calculation of the dielectric tensor using Equations (2)–(5), we obtained the absorption and reflectivity for each structure. We considered the propagation of the electromagnetic wave perpendicular to the graphene plane. In Figure 9, Figure 10 and Figure 11 the absorption reported in the *y*-axis is expressed in eV. We used the fact that a wave with a wavenumber *k* = 8065.544 cm^−1^ would correspond with a photon with an energy of 1 eV.

#### 2.4.1. Infrared

We obtained the principal infrared absorption peak for graphene with 5.55% vacancies extinguished upon interaction with any of the four amino acids studied, as shown in Figure 10. This peak was in the absorption band of amide I at 1690 cm^−1^ or 0.20953 eV [21], so graphene with 5.55% vacancies could function as a sensor for this bond.

When doped with fluorine, only glycine left the IR absorption spectrum unchanged. In contrast, the other three amino acids showed a peak at 0.70 eV. This variation could already be detected because the higher energy resolution of the sensors could be around 0.0067 eV [22]. With this graphene doped to 5.55%, fluorine could differentiate glycine from leucine, proline, or alanine. If we doped with nitrogen, the spectrum did not change enough to be detected because the variations found were less than 0.0067 eV. Proline could be detected in the IR spectrum with oxygen-doped graphene at 5.55% and clearly differed from alanine, glycine, and leucine. The peak was located at 1.12 eV.

#### 2.4.2. Visible

Figure 11 shows the variations in the visible spectrum. Both glycine and leucine increased the absorption in the blue (2.70 eV) in graphene with vacancies. This could make such a modified graphene surface a good detector for both amino acids. In this same interval, both glycine and leucine could be selectively detected from alanine and proline.

For the F-doped graphene case, glycine was the only amino acid that kept the absorption spectrum unchanged. The other three amino acids increased the activity in both orange (2.02 eV) and violet (3.15 eV), while cancelling the peak generated when doped with fluorine. The differences obtained would be enough to be detected, considering that current biosensors can detect differences of around 0.0067 eV [22]. For the N-doped graphene, proline should have been detected in the orange (2.23 eV) and blue (2.70 eV) region. It was hard to differentiate between the other three amino acids in this region.

#### 2.4.3. Ultraviolet

**Figure 12:**
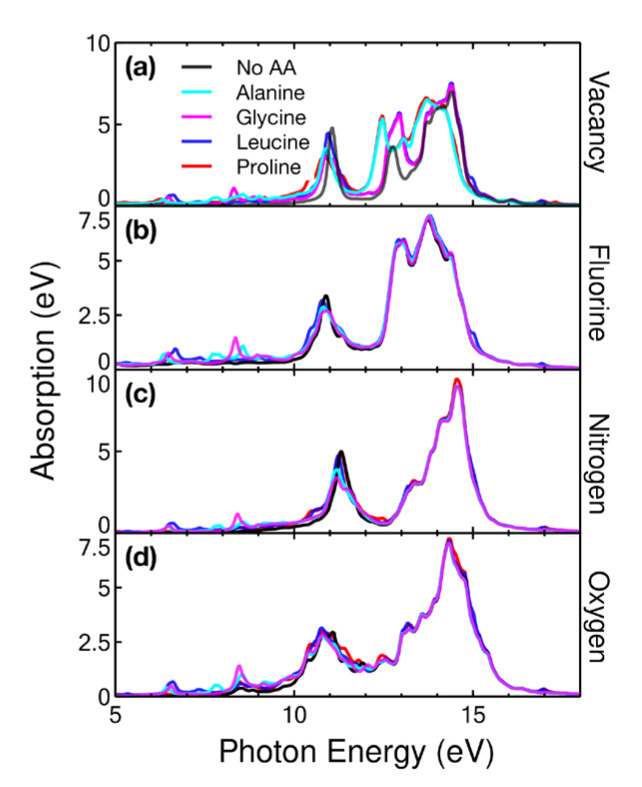
Absorption in the ultraviolet region for the cases considered: (**a**) vacancy, (**b**) F-doped, (**c**) N-doped, and (**d**) O-doped graphene at a concentration of 5.55% interacting with alanine, glycine, leucine, and proline.

In Figure 12, we show the absorption in the ultraviolet region. Pristine graphene had two prominent peaks in this range; the first corresponded with incident radiation, with an energy of 11.02 eV, and the second, of 14.58 eV. When vacancies were added to graphene at 5.55%, a broadening of the second peak occurred and a third peak appeared at 12.8 eV. The effect of proline and alanine was practically the same, with both increasing the UV absorption in the range of 12.5 to 14.5 eV. Glycine and leucine only increased and shifted the 12.6 eV peak to the right.

The effect of the four amino acids on the three doped-graphene cases considered was negligible for the main peaks. Glycine produced a peak at 8.5 eV in the four modified graphene cases. The four amino acids considered caused a small amount of UV absorption, with an energy of 6.5 eV.

#### 2.4.4. Reflectivity

**Figure 13:**
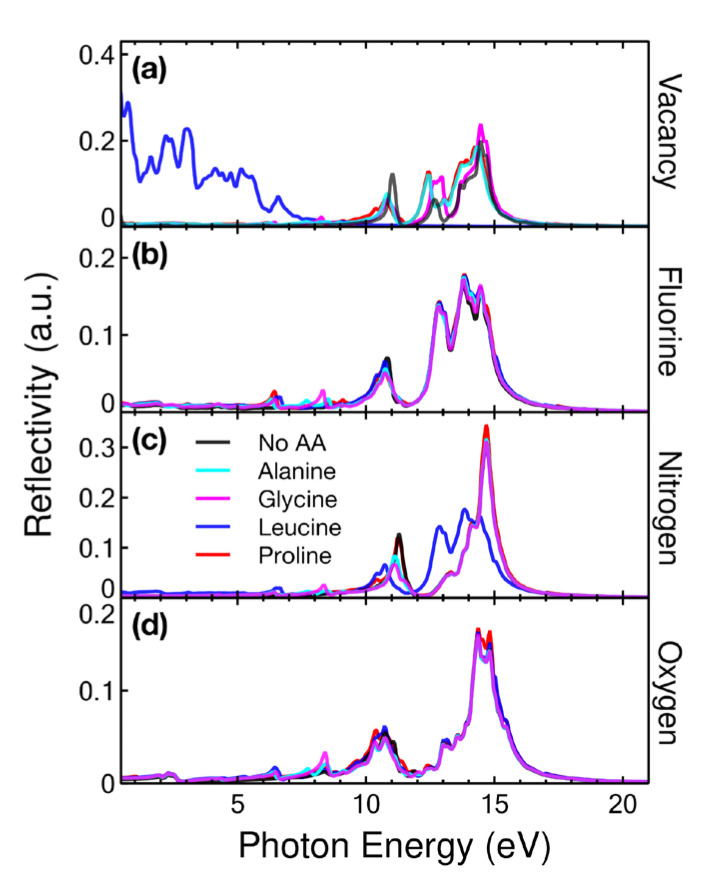
Reflectivity for the cases considered: (**a**) vacancy, (**b**) F-doped, (**c**) N-doped, and (**d**) O-doped graphene at a concentration of 5.55% interacting with alanine, glycine, leucine, and proline.

Reflectivity was calculated over a range from 0 to 30 eV and plotted up to 20 eV because from this energy, the reflectivity became zero in all cases. Figure 13a shows the most important change when graphene with 5.55% vacancies interacted with Leu. The two main peaks became zero and graphene showed activity in the region below 8 eV.

The other amino acids only produced an increase in the peaks at 12 and 14 eV, while the peak at 11 eV decreased. When graphene was doped with F, N, or O, the connectivity remained invariant when interacting with alanine or glycine. Only a small peak was seen for both cases at an energy of 8.30 eV.

## 3. Discussion

In this work, we performed ab initio DFT calculations to study the electrical and optical properties of modified graphene and its interactions with four amino acids (alanine, glycine, leucine, or proline), which can be considered to be the building blocks of spider silk. Four cases of graphene modifications were considered: graphene with vacancies at 5.55% and fluorine-, oxygen-, or nitrogen-doping of graphene at the same percentage. For all cases, we found that the electronic properties—the projected density of states, band structure, and Löwdin charges—overall remained unchanged while interacting with the four amino acids considered. Regarding the recovery time, F-doped graphene at 5.55% could be the most suitable proline detector among the cases considered.

Graphene with vacancies could also be a suitable glycine sensor, even if it presents an adsorption energy 0.08 eV higher than the detection upper limit. A higher adsorption energy could saturate the sensor, while a lower energy would not be enough to detect the amino acid.

On the other hand, there were interesting findings on the optical properties of the systems considered. We found that the case of graphene with vacancies presented the adsorption of amino acids, with proline having the highest adsorption energy (2.24 eV). A loss of the peak in the infrared spectrum (0.20 eV) when interacting with the four amino acids was also found. Thus, the vacancy percentage considered (5.55%) could be used to detect amide. For this case, we also found blue light absorption when interacting with glycine and leucine, differentiating them from alanine and proline.

In addition, we obtained a cancellation of the three peaks of the reflectivity of the graphene with vacancies. This case showed an activity of 20% in the visible region when interacting with leucine, making it a possible optical sensor in the said region.

Fluorine-doped graphene at 5.55% could also be used to differentiate between alanine and glycine by absorption in the orange range. We found that N- and O-doped graphene required a higher resolution to differentiate between amino acids, which could also imply a cost increment of a sensor based on those surfaces.

## 4. Materials and Methods

The ab initio calculations performed in this work were obtained using the density functional theory (DFT) and the software suite Quantum ESPRESSO ver. 6.2.1 [23,24]. XCrySDen software ver. 1.6.2, was used for visualization purposes [25] and Veusz ver. 3.4 for graphs [26]. We considered the pseudopotential formalism and a plane wave approximation with an energy cut-off value of 80 Ry. In calculating the band structure, the projected density of states (PDOS), and the dielectric tensor, we used the generalized gradient approximation (GGA) method and the Perdew–Burke–Ernzerhof (PBE) expression for the exchange–correlation functional [27].

We also considered norm-conserving pseudopotentials with the Goedecker–Hartwigsen–Hutter–Teter method and the Van der Waals VdW–DFT correction [28,29,30,31] as well as a 8 × 8 × 2 k-point mesh within the Monkhorst–Pack scheme [32]. The energy convergence threshold for self-consistency was set to 10^−8^ Ry. As the code used considers periodic boundary conditions, the supercell size was large enough to avoid spurious interactions. For the same reason, the cell parameter in the *z*-axis was set to 15 Å, meaning there was a 30 Å distance between each graphene layer. In this way, we could model a 2D surface in a vacuum.

We first optimized the graphene surface for the four cases considered: addition of vacancies, and F-, N-, or O-doping. Then, the amino acids were included. We performed a geometric optimization to minimize the forces of the system below a threshold of 1.0 × 10^−3^ Ry/Bohr. Self-consistent and non-self-consistent calculations were performed on the resultant structures to obtain their corresponding band structures, the density of states, and the PDOS.

In each case, the imaginary part of the dielectric tensor (Im *ε_ii_*(*ω*)) was calculated from the band structure. Then, the Kramers–Kroning relation was used to calculate the real part of the dielectric tensor (Re *ε_ii_*(*ω*)). Knowing the real and imaginary part of the dielectric tensor, the reflectivity (*R_ii_*(*ω*)) and optical absorption (*A_ii_*(*ω*)) were obtained via the following equations [33]:

(2) $$R_{ii}\left(\omega \right)=\frac{{\left(\;n\;-1\right)}^{2}{+k}^{2}}{{\left(n+1\right)}^{2}{+k}^{2}}$$

(3) $$A_{ii}\left(\omega \right)=\frac{2\omega k\left(\omega \right)}{c}$$

where

(4) nii=εiiω+Reεiiω2 

(5) kiiω=εiiω−Reεiiω2

In Equations (2)–(5), *n* is the refractive index and *k* is the extinction coefficient. Finally, considering Eyring’s theory of state transitions [19,20], we calculated the recovery times, which allowed us to estimate the absorption time of the amino acid onto the surface. These were obtained with the following equation:

(6) $$\tau =\left(\frac{h}{K_{B}T}\right)e^{-E_{\mathrm{ads}}{/K}_{B}T}$$

In this expression, *E*_ads_ is the adsorption energy of each molecule within the surface and *K_B_* and *h* are Boltzmann’s and Plank’s constants, respectively. Finally, *T* is the temperature, set here to be 300 K. We did not perform molecular dynamics calculations in this work; the value of *T* = 300 K was exclusively used in Equation (6) to estimate the recovery time at room temperature.

## Figures and Tables

**Table 1:** Adsorption energies (in eV) for each modified graphene structure interacting with alanine, glycine, leucine, and proline.

Amino Acid	Vacancy	Fluorine	Oxygen	Nitrogen
Alanine	−1.94	−0.09	−0.20	−0.26
Glycine	−0.90	−0.02	−0.01	−0.21
Leucine	−1.04	−0.20	−0.40	−0.42
Proline	−2.24	−0.65	−0.55	−0.57

**Table 2:** Recovery times (in seconds) for each modified graphene structure interacting with alanine, glycine, leucine, and proline.

Amino Acid	Vacancy	Fluorine	Oxygen	Nitrogen
Alanine	6.01 × 10^19^	5.21 × 10^−12^	3.55 × 10^−9^	4.17 × 10^−10^
Glycine	2.22 × 10^2^	4.15 × 10^−13^	4.62 × 10^−10^	2.59 × 10^−13^
Leucine	4.71 × 10^4^	6.01 × 10^−10^	1.52 × 10^−6^	9.71 × 10^−7^
Proline	5.73 × 10^24^	1.26 × 10^−2^	6.88 × 10^−4^	3.29 × 10^−4^

## Data Availability

Data is unavailable due to privacy restrictions.
